# Anemia before in-hospital cardiac arrest and survival from cardio-pulmonary resuscitation—a retrospective cohort study

**DOI:** 10.1186/s44158-022-00080-5

**Published:** 2022-12-20

**Authors:** Lior Shor, Yigal Helviz, Sharon Einav

**Affiliations:** 1 Department of Military Medicine and “Tzameret”, Faculty of Medicine, Hebrew University of Jerusalem, Jerusalem, Israel; 2 Medical Corps, Israel Defense Forces, Jerusalem, Israel; 3 General Intensive Care Unit, Shaare Zedek Medical Center, 12 Shmuel Bait St, PO Box 3235, 9103102 Jerusalem, Israel; 4 Faculty of Medicine, Hebrew University, Jerusalem, Israel

**Keywords:** Cardiopulmonary resuscitation, Heart arrest, Anemia, Comorbidity, Survival

## Abstract

**Background:**

Multiple patient-related variables have been associated with reduced rates of survival to hospital discharge (SHD) after in-hospital cardiac arrest (IHCA). As opposed to most of these, anemia may be reversible. This retrospective single-center study aims to examine the relationship between prearrest hemoglobin levels, comorbidities, and survival after cardiopulmonary resuscitation (CPR) among patients with non-traumatic IHCA. Patients were classified as anemic (hemoglobin < 10 g/dL) or non-anemic (hemoglobin ≥ 10 g/dL) based on their lowest hemoglobin measurement in the 48 h preceding the arrest. The primary outcome was SHD. The secondary outcome was return of spontaneous circulation (ROSC).

**Results:**

Of 1515 CPR reports screened, 773 patients were included. Half of the patients (50.5%, 390) were classified as anemic. Anemic patients had higher Charlson Comorbidity Indices (CCIs), less cardiac causes, and more metabolic causes for the arrest. An inverse association was found between CCI and lowest hemoglobin. Overall, 9.1% (70 patients) achieved SHD and 49.5% (383) achieved ROSC. Similar rates of SHD (7.3 vs. 10.7%, *p* = 0.118) and ROSC (49.5 vs. 51.0%, *p* = 0.688) were observed in anemic and non-anemic patients. These findings remained consistent after adjustment for comorbidities, in sensitivity analyses on the independent variable (i.e., hemoglobin) and on potential confounders and in subgroups based on sex or blood transfusion in the 72 h preceding the arrest.

**Conclusions:**

Prearrest hemoglobin levels lower than 10 g/dL were not associated with lower rates of SHD or ROSC in IHCA patients after controlling for comorbidities. Further studies are required to confirm our findings and to establish whether post-arrest hemoglobin levels reflect the severity of the inflammatory post-resuscitation processes.

**Supplementary Information:**

The online version contains supplementary material available at 10.1186/s44158-022-00080-5.

## Background

In-hospital cardiac arrest (IHCA) occurs in approximately 1.6:1000 hospital admissions [1]. The rates of survival from cardiac arrest are low (13.4% at 1 year) [2] and survivors have multiple complications [3, 4]. Multiple patient-related variables have been associated with reduced rates of survival to hospital discharge (SHD) after CPR (cardio-pulmonary resuscitation), including female sex [5], older age, comorbidities, and more [6]. As opposed to most of these patient-related variables, anemia may be reversible. However, it would require the use of blood products which may result in complications [7]. As a prospective study on this topic may be costly in terms of funding and potential complications, more data is required to determine whether such a study is justifiable in patients with cardiac arrest and if so, what cutoff values should be studied.

Few studies regarding the relationship between anemia and survival after CPR exist. These studies mostly associated lower hemoglobin levels with a lower likelihood of SHD [8, 9] and neurologically intact survival after CPR [8–14] (supplement 1). Correspondingly, anemia seems to be associated with poorer outcomes in various diseases states (e.g., heart failure [15], diabetes [16], malignancy [17]). Meanwhile, patients with an overall higher burden of disease, i.e., higher Charlson comorbidity score (CCI) [18, 19] and specific chronic diseases, e.g., heart failure, renal disease [20], and malignancy [19] have poorer survival outcomes after CPR. A conundrum therefore remains, whether the presence of anemia itself or the presence of comorbidities associated with anemia explains the association with poorer outcomes in anemic patients after CPR.

This study aimed to examine the relationship between prearrest hemoglobin levels and survival after CPR.

## Methods

### Study design

Ethical approval was waived for this single center study within the framework of a resuscitation quality assurance project (waiver P4.15, 18-Jan-2015). The findings of this cohort study are reported in accordance with STROBE (Strengthening the Reporting of Observational Studies) requirements [21].

### Setting

We retrospectively analyzed observational data collected in real time over a period of 4 years (2016–2019) in a tertiary teaching hospital (the Shaare Zedek Medical Center, SZMC). The SZMC staff undergo routine periodic accreditation in CPR as required by the Joint Commission Resources (JCR). CPR is conducted in accordance with International Liaison Committee of Resuscitation (ILCOR) recommendations. Resuscitation events are mandatorily reported on a standardized electronic sheet embedded within the patient electronic medical records (EMRs) (supplement 2) which conforms to Utstein reporting recommendations.

### Participants (eligibility criteria)

All IHCA reports for patients aged 18 years or over (Jan 2016–Dec 2019) were screened for inclusion. Reports of traumatic cardiac arrest, cardiac arrest during pregnancy or the peripartum period, and reports for patients without a single hemoglobin measurement in the 48 h before cardiac arrest were excluded. Also excluded were reports with hemoglobin measurements that did not pass logical testing (below 4 g/dL or above 20 g/dL) and reports of repeat resuscitations (supplement 3). Intraoperative arrests were a priori not included in this study.

Based on prior literature (section “Management of quantitative variables”) the patients were divided into two groups: anemic (hemoglobin < 10 g/dL) and non-anemic (hemoglobin ≥ 10 g/dL).

### Study outcomes

The primary outcome was the association between prearrest anemia (< 10 g/dL) in the 48 h preceding the event and SHD before and after adjusting for background diseases. The secondary outcome was the association between prearrest anemia and ROSC before and after adjustment. We hypothesized that prearrest hemoglobin levels would be statistically related to unadjusted survival rates and that this association would disappear after adjustment for comorbidities.

### Variables

Among other variables we collected, patient demographics (e.g., age, sex), burden of comorbidity (specific diseases, CCI), hemoglobin levels, and data on prearrest blood transfusion (supplement 4).

Because missing data are major concern in retrospective studies, we collected two values of hemoglobin, the last value before the arrest and the lowest value in the 48 h preceding the arrest, and used the value with the least amount of missing data is for the main analysis.

We analyzed hemoglobin in three manners: dichotomized to anemic and non-anemic patients (primary analysis), as a continuous variable and divided to tertiles (secondary analyses).

### Method and sources of data collection

All data were extracted from the hospital CPR database and patient EMRs. The database algorithm collects data on patient comorbidities using the ICD-9 codes listed in the patients EMRs (supplement 5). For liver disease, renal failure, malignancy, and cerebrovascular disease, we determined disease severity—which is required for calculating the CCI [18]—case-by-case through manual evaluation of the full patient file. The data validation process is described in supplement 6.

### Address of bias

Reporting bias is always a possibility; however, our overall rate of SHD is similar to that described elsewhere [2, 22]. Selection bias stemming from a greater likelihood of measuring hemoglobin in patients who were in a worse clinical condition was dealt with by studying hemoglobin levels both 24 and 48 h before the event. Patients with no relevant measurement comprised less than 10% of eligible cases even before reduction of repeat resuscitations (supplement 7). Hemoglobin is measured using three devices in our hospital; therefore, a systematic measurement bias may be possible. However, our devices undergo testing for miss-calibration in two manners; (1) monthly external quality assurance by the UK National External Quality Assessment Service and (2) daily internal quality assurance and harmonization between all three devices by the laboratory staff.

### Sample size

We calculated the study power for the primary analysis—difference in survival among anemic versus non-anemic (< 10 g/dL vs ≥ 10 g/dL) patients. Based on preliminary evaluation of the existing database we assumed at least 350 patients in each group. We assumed a survival rate of 25% among non-anemic patients versus 10% among anemic patients based on previously reported survival rates [22] and odds ratios for survival [10]. With a two-sided significance level of 5%, the study power would be > 99%. We also calculated that the study power would be 96% with half the sample size and 83% with a lower difference in survival rates (8 vs 15%).

### Management of quantitative variables

For the definition of anemia, we selected a-priori a cutoff of 10 g/dL based on previous studies that found an association between hemoglobin and CPR outcomes [8, 10, 11] and on the accepted maximal transfusion threshold for patients with cerebral and/or cardiac ischemia [23, 24]. The World Health Organization criteria for anemia differ for men and women [25]. We nonetheless chose a uniform threshold since no such differentiation is usually made for critically ill patients [8–14, 26].

We used a modified version CCI as follows: points for peptic ulcer disease were added for a proven diagnosis rather than based on receipt of therapy due to increased use of preventive treatment since the CCI was published [27]. In addition, points were not added for age. We examined age as a standalone variable since it is strongly related to both comorbidities and survival [28]. Furthermore, points for age were only added to the later iteration of the CCI [29]. Other than these two caveats, the score added per comorbidity adheres to the standard [18].

### Statistical methods

We used descriptive statistics to assess demographics, comorbidities, and other patients’ characteristics. All proportions were calculated from the total number of patients (including those with missing data). We report all summary measures regardless of variable distribution.

Categorical variables were compared using the chi-square or Fisher’s exact tests, and continuous variables were compared using the *T* test or one-way analysis of variance, as required. Correlations were studied using the Pearson correlation coefficient. All *p* values were two-sided (when applicable), and confidence intervals were computed at the 95% confidence level.

Receiver operating characteristic (ROC) analysis was used to test the a priori chosen cutoff value used to define anemia (10 g/dL) against other possible cutoff values found using Liu’s method [30].

Multivariable logistic regression analysis (enter method) was used in order to simultaneously assess the effect of several independent factors on the dichotomous dependent outcomes (ROSC or SHD). The threshold for inclusion in the multivariable model was a *p* value ≤ 0.1. Multicollinearities were sought using clinical logic and confirmed using the chi-square test. In case of strong multicollinearity, we examined the effect of excluding each variable on the models and chose the models with the best goodness-of-fit. An initial shockable rhythm was highly correlated with shock delivery and coronary angiography was highly correlated with coronary reperfusion attempt. We therefore excluded shock delivery and coronary reperfusion attempt from the models.

In order to study model stability, we performed sensitivity analysis on the definition of the independent variable, on potential confounders and on post-CPR coronary angiography (in order to examine the effect of the treatment while retaining in the model patients that did not achieve ROSC).

We also conducted post hoc analyses to test for association between anemia and survival outcomes (SHD and ROSC) for three subgroups: males, females, and patients who had not received a blood transfusion within the 72 h before CPR.

All analyses were performed using SPSS version 26.0 (IBM corp., Armonk, NY, USA). [31].

## Results

**Fig. 1:**
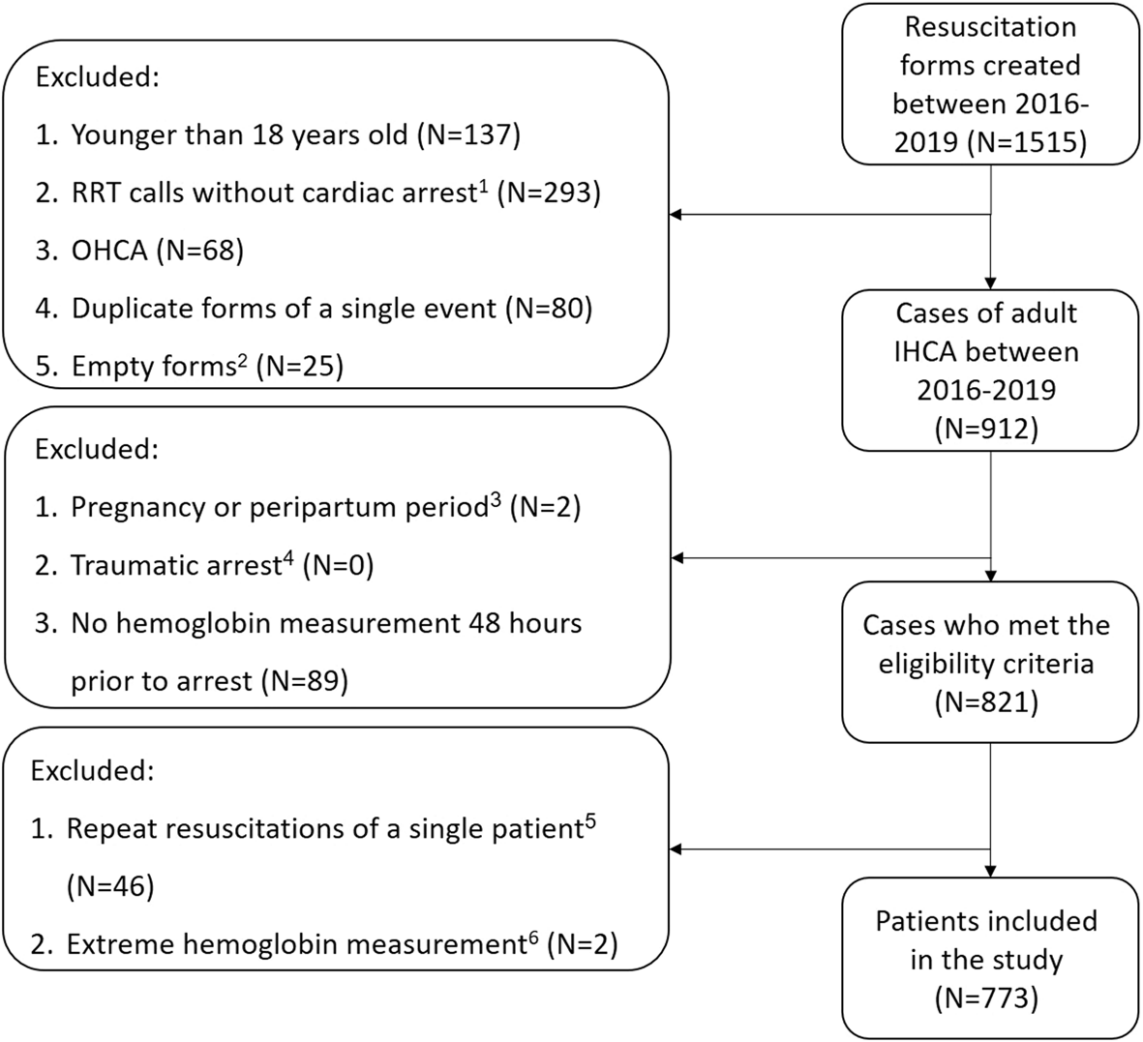
Study inclusion exclusion flow chart. RRT rapid response team. OHCA out-of-hospital cardiac arrest. ROSC return of spontaneous circulation. ^1^Ascertained either by manual evaluation or by a statement in the electronic form that cardiac arrest had not occurred and no evidence of chest compressions was found. ^2^No information entered to the electronic form by the on-site medical team and therefore could not be verified. ^3^Ascertained by excluding events reported from the delivery room, maternity wards, or department of gynecology. ^4^Ascertained by manual review of cases reported from the ED, general surgery, operating theatre, postoperative care unit, or any location in the imaging. ^5^In case of repeat resuscitations of a single patient, only the first arrest was included. ^6^Hemoglobin measurements lower than 4 g/dL or higher than 20 g/dL

Overall, 1515 reports were screened. At the end of the inclusion–exclusion process 773 patients were included (Fig. 1). We hereby describe mostly results based on the lowest hemoglobin measurements (primary analysis). More results based on the last hemoglobin measurement are available in supplement 8.

### Cohort characteristics

The mean age was 76.58 ± 14.21 years and 55.8% were male. The mean CCI was 2.07 ± 1.91. The mean lowest hemoglobin level was 10.27 ± 2.31 g/dL (median 9.9 g/dL, IQR 8.5–11.8 g/dL, range 4.6–19.2 g/dL). Lowest hemoglobin tertiles cutoffs were 1st: ≤ 8.9 g/dL, 2nd: > 9.0 g/dL to ≤ 11.1 g/dL, and 3rd: > 11.1 g/dL. For hemoglobin distribution, see supplement 9. 49.5% (383) achieved ROSC and 9.1% (70) survived to hospital discharge (Tables 1 and 2).

**Table 1:** Patients’ characteristics and comorbidities. Anemia was defined based on the lowest hemoglobin in the 48 h preceding the arrest—categorial variables. Values are presents as *n* (%)

	Total (*n* = 773)	No anemia (*n* = 383)	Anemia (*n* = 390)	*P* Value
Characteristics				
Male sex	431 (55.8)	207 (54)	224 (57.4)	0.343
Cause of arrest				
Cardiac	303 (39.2)	165 (43.1)	138 (35.4)	0.024
Respiratory	300 (38.8)	142 (37.1)	158 (40.5)	
Metabolic	25 (3.2)	5 (1.3)	20 (5.1)	
Neurologic	9 (1.2)	4 (1)	5 (1.3)	
Infection	76 (9.8)	40 (10.4)	36 (9.2)	
Other	23 (3)	10 (2.6)	13 (3.3)	
Initial shockable rhythm	78 (10.1)	48 (12.5)	30 (7.7)	0.027
Blood transfusion 72 h prior to CPR	99 (12.8)	11 (2.9)	88 (22.6)	< 0.001
Event witnessed	431 (55.8)	224 (58.5)	207 (53.1)	0.086
Chest compressions	763 (98.7)	380 (99.2)	383 (98.2)	0.505
Shock delivered	188 (24.3)	99 (25.8)	89 (22.8)	0.326
Lidocaine given	18 (2.3)	10 (2.6)	8 (2.1)	0.589
Amiodarone given	85 (11)	45 (11.7)	40 (10.3)	0.471
ROSC	383 (49.5)	188 (49.1)	195 (50)	0.799
ECPR	10 (1.3)	7 (1.8)	3 (0.8)	0.213
TTM	2 (0.3)	1 (0.3)	1 (0.3)	1.000
Coronary angiography	26 (3.4)	20 (5.2)	6 (1.5)	0.003
Coronary reperfusion attempt	20 (2.6)	15 (3.9)	5 (1.3)	0.017
Survival to discharge	70 (9.1)	40 (10.4)	30 (7.7)	0.183
Discharge destination				
Home	25 (3.2)	18 (4.7)	7 (1.8)	0.082
Rehabilitation	17 (2.2)	10 (2.6)	7 (1.8)	
Nursing home	4 (0.5)	3 (0.8)	1 (0.3)	
Ventilated ward	19 (2.5)	7 (1.8)	12 (3.1)	
Deceased	703 (90.9)	343 (89.6)	360 (92.3)	
Comorbidities				
Acute MI	98 (12.7)	52 (13.6)	46 (11.8)	0.457
Old MI	38 (4.9)	22 (5.7)	16 (4.1)	0.291
CHF	289 (37.4)	151 (39.4)	138 (35.4)	0.246
PVD	22 (2.8)	10 (2.6)	12 (3.1)	0.697
Acute CVA	35 (4.5)	24 (6.3)	11 (2.8)	0.021
History of CVA	107 (13.8)	51 (13.3)	56 (14.4)	0.675
Dementia	57 (7.4)	37 (9.7)	20 (5.1)	0.016
Pulmonary disease	73 (9.4)	40 (10.4)	33 (8.5)	0.346
Connective tissue disease	6 (0.8)	4 (1)	2 (0.5)	0.447
Gastrointestinal ulcer	8 (1)	4 (1)	4 (1)	1.000
Liver disease	16 (2.1)	5 (1.3)	11 (2.8)	0.139
Diabetes mellitus	309 (40)	138 (36)	171 (43.8)	0.027
Paraplegia	0 (0)	0 (0)	0 (0)	
Renal failure	148 (19.1)	57 (14.9)	91 (23.3)	0.003
Solid tumors	119 (15.4)	49 (12.8)	70 (17.9)	0.047
Hematologic malignancy	26 (3.4)	7 (1.8)	19 (4.9)	0.019
AIDS	0 (0)	0 (0)	0 (0)	

*CPR* cardio-pulmonary resuscitation, *ROSC* return of spontaneous circulation, *ECPR* extracorporeal cardiopulmonary resuscitation, *TTM* targeted temperature management, *MI* myocardial infarction, *CHF* congestive heart failure, *PVD* peripheral vascular disease, *CVA* cerebrovascular accident, *AIDS* acquired immune deficiency syndrome

Missing data—cause of arrest–37 (4.8%), initial shockable rhythm–32 (4.1%), event witnessed–20 (2.6%), chest compressions–1 (0.1%), shock delivered–2 (0.3%), lidocaine and amiodarone–26 (3.4%), ECPR, TTM, coronary angiography, and coronary reperfusion attempt–390 (50.5%), discharge destination–5 (0.6%)

**Table 2:** Patients’ characteristics. Anemia was defined based on the lowest** hemoglobin in the 48 h preceding the arrest**—continuous variables

Characteristics	Total (*n* = 773)			No anemia (*n* = 383)			Anemia (*n* = 390)			P-Value
	Mean ± SD	Median (IQR)	Range	Mean ± SD	Median (IQR)	Range	Mean ± SD	Median (IQR)	Range	
Age (years)	76.58 ± 14.21	80 (68–86)	18–106	77.52 ± 14.69	81 (69–88)	19–106	75.66 ± 13.68	78 (68–85)	18–101	0.069
CCI	2.07 ± 1.91	2 (1–3)	0–11	1.86 ± 1.84	2 (1–3)	0–10	2.28 ± 1.96	2 (1–3)	0–11	0.002
Adrenaline (mg)	5.38 ± 4.08	5 (3–7)	0–36	5.8 ± 4.38	5 (3–7)	0–36	4.97 ± 3.73	4 (3–6)	0–35	0.007

*CCI* Charlson comorbidity index, *SD* standard deviation, *IQR* interquartile range

Missing data–adrenaline—43 (5.6%)

Based on the lowest hemoglobin measurement, half of the patients (50.5%, *n* = 390) were classified as anemic. Anemic patients had a higher prevalence of diabetes, renal failure, solid tumors and hematologic malignancies, and a lower prevalence of dementia and acute CVA. Anemic patients had a higher overall burden of disease (i.e., higher CCIs) and different causes for arrest. More anemic patients received a blood transfusion in the 72 h preceding the arrest. Less anemic patients had an initial shockable rhythm and anemic patients received less adrenaline (epinephrine). Less anemic patients underwent coronary angiography and a coronary reperfusion attempt after CPR (Tables 1 and 2).

### Lowest hemoglobin and Charlson comorbidity index

**Fig. 2:**
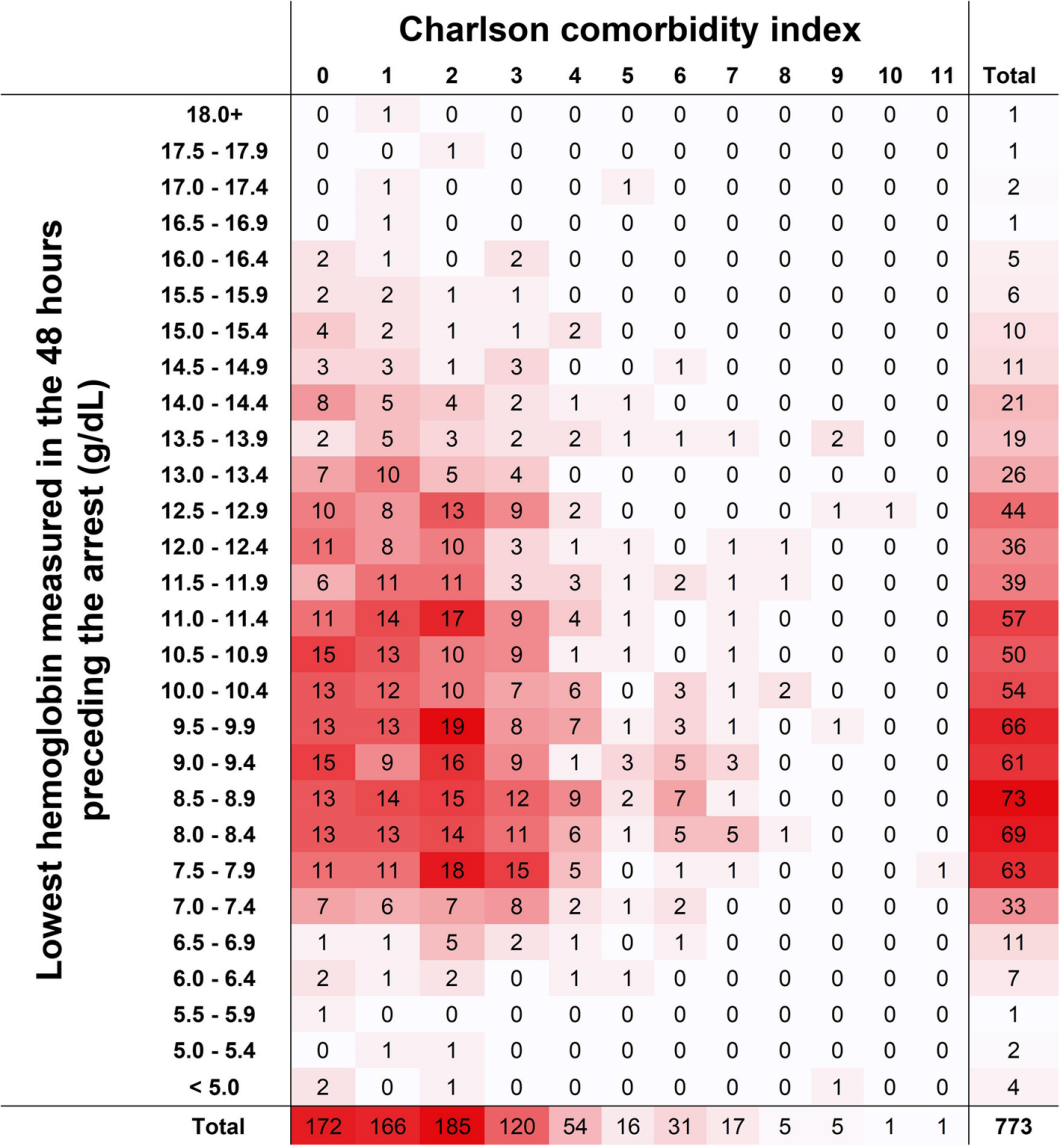
Heat map of Charlson comorbidity index versus lowest hemoglobin measured in the 48 h preceding the arrest (g/dL). Heat map showing the distribution of hemoglobin measurements between CCI scores highlighting that with lower hemoglobin measurements CCI rise

An inverse association was found between CCI and lowest hemoglobin; heat maps of the relationship between CCI and hemoglobin show that as CCIs rise, hemoglobin measurements are lower (Fig. 2).

We studied this association in three manners: First by comparing mean CCI in anemic and non-anemic patients (2.28 ± 1.96 vs 1.86 ± 1.84, *p* = 0.002), then by comparing the mean CCI in three groups of hemoglobin tertiles (1st: 2.32 ± 1.96, 2nd: 2.02 ± 1.86, and 3rd: 1.87 ± 1.89, *p* = 0.022) and finally, using CCI and hemoglobin as continuous variables (*p* value 0.005, Pearson’s *r* − 0.100) (supplement 10a).

### Survival to hospital discharge

#### ROC analysis

**Fig. 3:**
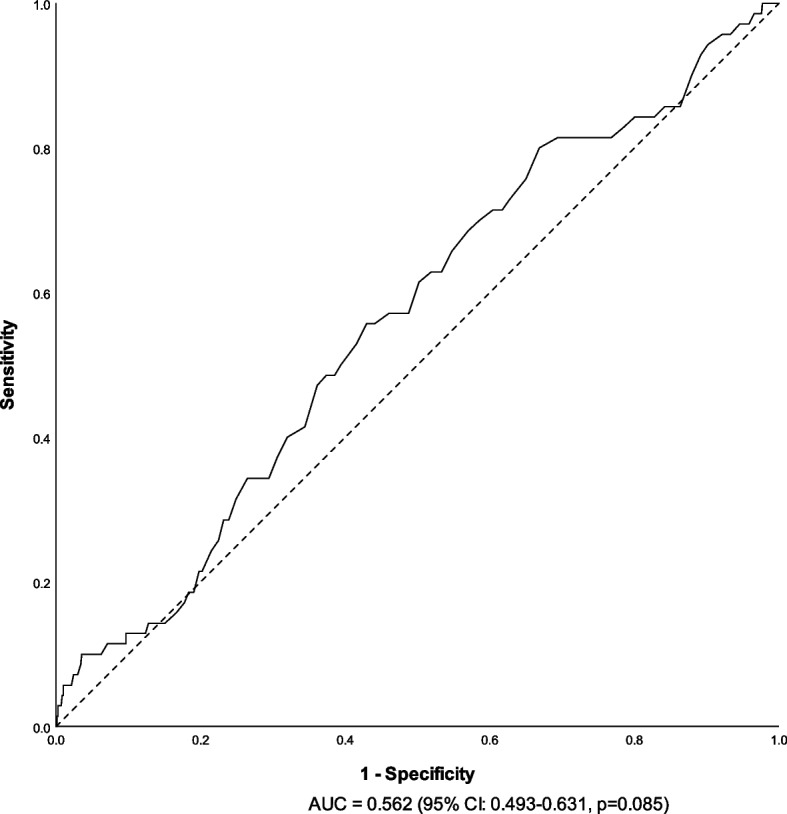
Receiver operating characteristic curve for prediction of survival to hospital discharge based on the lowest hemoglobin concentration measured in the 48 h preceding the arrest. AUC area under the curve, CI confidence interval

ROC analysis (Fig. 3) highlighted a cutoff of 10.35 g/dL with sensitivity and specificity of 0.557 and 0.570 compared to 0.571 and 0.526 for 10 g/dL. Due to the comparability between the cut-offs, we proceeded with the a priori chosen cutoff (10 g/dL).

#### Univariable analysis

No unadjusted association was found between the lowest hemoglobin and SHD. We studied this association in three manners: First by comparing survival rates in anemic vs. non-anemic patients (7.7 vs 10.4%, *p* = 0.183) (primary outcome), then by comparing survival rates in the three hemoglobin tertiles (1st: 6.5%, 2nd: 9.7%, and 3rd: 11.1%, *p* = 0.171) and by comparing hemoglobin levels in survivors and non-survivors (10.8 ± 2.4 g/dL vs 10.2 ± 2.3 g/dL, *p* = 0.055) (supplement 11).

We did find an unadjusted association between last hemoglobin and SHD (11.2 ± 2.5 g/dL in survivors vs 10.6 ± 2.3 g/dL in non-survivors, *p* = 0.042) (supplement 8).

Other factors found associated with decreased SHD in univariable analysis were older age, solid tumors, higher CCI, initial non-shockable rhythm, no shock administration (not included in the models), higher adrenaline dose, and no post-CPR coronary angiography or reperfusion attempt (also not included) (supplements 11,12).

#### Multivariable analysis

The primary multivariable analysis included 348 patients. CCI, an initial shockable rhythm, lower adrenaline dose and coronary angiography remained associated with SHD in the adjusted analysis. Neither age nor anemia based on the lowest hemoglobin (adjusted OR: 0.701, CI: 0.363–1.352, p = 0.289) were associated with SHD in the adjusted analysis (Table 3, model 1).

**Table 3:** Multivariable analysis for survival to hospital discharge (OR > 1). The independent factor is the dichotomized variable, anemia **based on the lowest hemoglobin in the 48 h preceding the arrest**

Model 1: excluding patients that did not achieve ROSC (in order to control for post-CPR treatments) *N* = 348, goodness o fit *p* value = 0.509						
Variable	Univariable			Multivariable		
	OR	95% CI	*P* value	OR	95% CI	*P* value
Age	0.970	0.956–0.984	< 0.001	0.998	0.978–1.017	0.819
CCI	0.839	0.719–0.979	0.026	0.741	0.592–0.927	0.009
Initial shockable rhythm	4.581	2.681–8.779	< 0.001	3.896	1.698–8.940	0.001
Adrenaline (mg)	0.663	0.575–0.764	< 0.001	0.664	0.561–0.784	< 0.001
Coronary angiography	2.569	1.094–6.033	0.035	7.720	2.302–25.897	0.001
Lowest hemoglobin anemia	0.715	0.435–1.173	0.183	0.701	0.363–1.352	0.289
Model 2: sensitivity analysis including patients that did not achieve ROSC (OR > 1) *N* = 700, goodness of fit *P* value = 0.342						
Variable	Univariable			Multivariable		
	OR	95% CI	*P* value	OR	95% CI	*P* value
Age	0.970	0.956–0.984	< 0.001	0.980	0.963–0.997	0.022
CCI	0.839	0.719–0.979	0.026	0.816	0.684–0.974	0.024
Initial shockable rhythm	4.581	2.681–8.779	< 0.001	5.478	2.610–11.497	< 0.001
Adrenaline (mg)	0.663	0.575–0.764	< 0.001	0.652	0.560–0.759	< 0.001
Lowest hemoglobin anemia	0.715	0.435–1.173	0.183	0.712	0.390–1.301	0.269

*OR* odds ratio, *CI* confidence interval, *CCI* Charlson comorbidity index

Anemia based on the last hemoglobin (adjusted OR 0.605, CI 0.294–1.245, *p* = 0.172) was also not associated with SHD in the adjusted analysis (supplement 8).

#### Sensitivity analyses

Sensitivity analysis on the independent variable (hemoglobin) as hemoglobin levels of survivors and non-survivors and hemoglobin tertiles showed no adjusted association with SHD.

Sensitivity analysis on the potential confounders (comorbidities) was performed by entering individual comorbidities that showed an unadjusted association with SHD into the multivariable analysis. Hence, solid tumors were entered. No adjusted association was seen between anemia and SHD.

Sensitivity analysis including patients that did not achieve ROSC showed no adjusted association between anemia and SHD (Table 3, model 2).

These results were consistent in a secondary analysis, based on last hemoglobin measurements (supplement 13).

#### Subgroup analyses

We found no unadjusted association between anemia and SHD in males, females, or patients that did not receive blood transfusion in the 72 h preceding their arrest (supplement 14).

#### ROSC

No unadjusted association was found between the lowest hemoglobin and ROSC when comparing ROSC rates in anemic and non-anemic patients (50.0 vs 49.1%, *p* = 0.799) (Table 1).

Anemia based on the lowest hemoglobin (adjusted OR 1.040, CI 0.746–1.450, *p* = 0.816) and CCI were not associated with ROSC after adjusting for age, blood transfusion, witnessed event, initial rhythm, and adrenaline dose (supplement 15).

## Discussion

This study showed that hemoglobin levels below 10 g/dL prior to cardiac arrest were not associated with lower rates of survival to hospital discharge or ROSC in IHCA patients. These results were consistent after controlling for comorbidities and in subgroup analyses done by sex and the subgroup of patients that had no blood transfusion prior to the arrest. Higher hemoglobin levels were found in survivors to hospital discharge; however, this association disappeared after controlling for comorbidities (i.e., CCI), demographics (i.e., age), event-related factors (i.e., initial shockable rhythm and adrenaline dose), and post-CPR treatments (i.e., coronary angiography after CPR). The consistency of the results, in addition to power of the study as calculated by the sample size calculation and previously reported proportions [10], should establish that such association is at the very least diminished in the setting of IHCA.

This study is unique due to its focus on IHCA patients and the measurement of pre-arrest hemoglobin levels. Moreover, the array of confounders studied and our approach to data analysis are also unique when comparing to the current literature on the topic.

The proportion of males, mean age, rates of initial shockable rhythm, and ROSC rates in our study are comparable to those in other studies on IHCA patients [1]. We also found similar comorbidity rates (e.g., diabetes mellitus, myocardial infarction, heart failure, acute CVA) to those described in other studies of IHCA patients [9, 10]. Although we studied pre-arrest hemoglobin levels, we found hemoglobin levels similar to those described in studies of post-ROSC hemoglobin in IHCA [9, 10]. The survival rates in our study are within the range described in other studies on IHCA patients [2, 22].

Our findings differ from those of prior studies on this topic in several aspects. Previous studies showed an association between hemoglobin and cardiac arrest outcomes even after controlling for comorbidities [8–10, 13, 14]. Our focus on IHCA patients may be one reason for this difference. Several previous studies controlled for individual comorbidities [8–10, 13, 14] and others did not control for any [11, 12]. We controlled for CCI which better represents the burden of comorbidity than individual comorbidities and also studied individual comorbidities separately. We included in the univariable analysis, patients that underwent CPR regardless of their ROSC status and performed a sensitivity analysis of the multivariable models by alternately excluding and including patients that did not achieve ROSC. Previous studies only excluded patients that did not achieve ROSC [8–14] and post-ROSC hemoglobin may be related to the severity of post cardiac arrest syndrome. Few patients received TTM in our study, most likely due to its controversial use in IHCA [32].

The results of our study support the findings of a recent study that suggested that in this context hemoglobin may act as a marker of other systemic processes; this study showed an association between increasing hemoglobin levels after cardiac arrest and worse outcomes [33]. The authors theorized that hemoglobin concentration may serve as a marker for the vascular permeability caused by post cardiac-arrest inflammation. If so, changes in hemoglobin levels may indicate the severity of the ongoing acute processes and a single value may be unrelated to survival despite its role in oxygen delivering capacity, as proposed in previous studies [14].

Our study has several limitations. The study was performed in a single center and may therefore not be generalizable. However, we show a similar case mix in terms of age [1] and background diseases [9, 10] as described in other studies on IHCA. Our data is highly dependent on the coding of ICD-9 diagnoses in the electronic medical charts. However, part of the work done for the purpose of this study was a quality assurance process to ensure code correctness. We present no data on neurological or functional outcomes yet these are considered the most meaningful outcomes after CPR. This decision stemmed from the small number of neurologically intact survivors expected in this single center and some lack of data.

In conclusion, pre-arrest hemoglobin levels lower than 10 g/dL were not associated with lower rates of survival to hospital discharge or ROSC in IHCA patients both before and after controlling for comorbidities. In this study, this finding is supported in both the dichotomous analysis and the continuous analysis (i.e., the ROC curve), suggesting that setting a simple numeric threshold for treatment of hemoglobin levels may not be relevant in this patient population. Our findings need corroboration in additional cohorts of IHCA patients. Studies seeking an association between anemia and neurological outcome should also be conducted in these patients. Finally, both laboratory and human studies are required to establish whether post resuscitation hemoglobin concentration is indeed a marker for the severity of vascular permeability caused by the severity of post cardiac-arrest inflammation.

## Supplementary Information

**Additional file 1.** 

## Data Availability

The datasets used and/or analyzed during the current study are available from the corresponding author on reasonable request.

## Abbreviations

CCI: Charlson comorbidity indices
CPR: Cardio pulmonary resuscitation
ICD-9: International Classification of Disease ninth revision
IHCA: Intra hospital cardiac arrest
ILCOR: International Liaison Committee of Resuscitation
JCR: Joint commission resources
ROSC: Return of spontaneous circulation
SHD: Survival to hospital discharge
STROBE: Strengthening the Reporting of Observational Studies
SZMC: Shaare Zedek Medical Center
TTM: Targeted temperature management
