# Supplementary material for: Anemia before in-hospital cardiac arrest and survival from cardio-pulmonary resuscitation—a retrospective cohort study
Source: J Anesth Analg Crit Care. 2022 Dec 20;2:51. doi: 10.1186/s44158-022-00080-5 (PMC10245541; doi:10.1186/s44158-022-00080-5)
Supplement: Supplementary file 1 — Additional file 1. [file 44158_2022_80_MOESM1_ESM.docx]

**The relationship between anemia prior to in-hospital cardiac arrest and survival from cardio-pulmonary resuscitation – a retrospective cohort study**

Lior Shor, Yigal Helviz, Sharon Einav

**Supplement Material**

**Supplement 1.** Summary table of studies regarding the association between hemoglobin and CPR outcome

| **Study ID, Design, Cases** | **Enrolment Period** | **Time of CPC Measurement** | **Time of Hemoglobin Measurement** | **Protocol Used** | **OHCA/ IHCA** | **Results** |
| --- | --- | --- | --- | --- | --- | --- |
| **Kim et al. 2018**^8^,  single center, 246 | 2009-2015 | hospital discharge | immediately after ROSC | TTM- only | OHCA | Higher Hb levels (OR 1.186, 95% CI 1.008-1.395, P<0.001) |
| **Wormsbecker et al. 2017**^9^, single center, 118 | 2009-2014 | hospital discharge | 48 h and 7 days following HIBI | TTM | IHCA+ OHCA | Lower 48h Hb levels (10 g/L unit OR 0.69, 95% CI 0.54-0.88, P<0.01) |
| **Johnson et al. 2016**^10^, multicenter, 598 | 2005-2011 | hospital discharge | 2 hours, 6  hours, 6-12 hours, 12-24 hours post-arrest. | TTM- only | IHCA+ OHCA | Higher Hb levels (OR 1.23, 95% CI 1.09-1.38, P<0.001) |
| **Albaeni et al. 2016**^11^, single center, 146 | 2004-2010 | hospital discharge | Post ROSC, every 6 h for the first 24 h and then every 24 h | ECMO, TTM | OHCA | Hb≥10g/dl (OR 8.31, 95% CI 1.89-36.52, P=0.005) |
| **Hayashida et al. 2014**^12^, multicenter, 495 | 2011-2013 | 90 days after CA | hospital arrival | TTM | OHCA | Higher cerebral oxy-Hb levels (unit OR:  1.60; 95% CI, 1.45 to 1.76; P < 0.001) |
| **SOS-KANTO. 2012**^13^, multicenter, 137 | 2002-2003 | 28 days after CA | Hospital arrival | TTM | OHCA | Higher Hb levels (OR, 1.26; 95% CI, 1.00-1.58, p=0.39) |
| **Wang et al. 2015**^14^, single center, 426 | 2006-2012 | hospital discharge | 24 h following ROSC | ECMO, TTM | IHCA | Higher product of HbXSpO_2_ (OR 1.003, 95% CI 1.002-1.004, P<0.001) |

All of the studies tested the association between hemoglobin levels post CPR and the neurological outcome. They all had a retrospective design (apart from Hayashida et al. 2014 which was conducted prospectively). A good neurological outcome was defined as CPC 1-2 in all of the studies.

CPR- Cardio-pulmonary resuscitation. CPC- Cerebral Performance Category. TTM- Targeted Temperature Management. CA- Cardiac Arrest. HIBI- Hypoxic Ischemic Brain Injury. ECMO- Extracorporeal Membrane Oxygenation. ROSC- Return of Spontaneous Circulation. OHCA/IHCA- Out/IN Hospital Cardiac Arrest. OR- Odds Ratio. CI- Confidence Interval. Hb- Hemoglobin. SpO2- peripheral capillary oxygen saturation.

**Supplement 2.** Clinical setting

The SZMC has more than 1000 admission beds, of which 850 are dedicated to adult inpatients. The department of emergency medicine (ED) is the largest in the district and receives approximately 150,000 referrals annually. Approximately 30-50 adult patients undergo CPR in the ED and 150-200 undergo CPR in other hospital wards each year.

The SZMC is a certified American Heart Association international training center and both training for- and performance of- CPR in the hospital are guideline-compliant. Protocolized targeted temperature management (TTM) has been used for more than 15 years and is set to <360C for patients with witnessed ventricular fibrillation (VF) or pulseless ventricular tachycardia (VT) that had remained unconscious subsequent to CPR. Coronary angiography is performed for patients diagnosed with cardiac ischemia.

Reporting follows Utstein recommendations; event data are documented in real time by the medical team on location using a designated electronic form. Post CPR follow-up is documented in the electronic medical chart and includes information on post resuscitation interventions (e.g., coronary angioplasty and survival status). Reports are validated and reviewed for internal consistency by a resuscitation nurse. Relevant case data are automatically updated in a CPR database which was constructed for audit and quality improvement purposes.

**Supplement 3.** Study inclusion exclusion ascertainment process

We defined traumatic arrest as patients who arrested up to six hours after admission for trauma and ascertained this by manual review of the files of patients who had arrested in the ED, general surgery, operating theatre, post-anesthesia care unit or any location in the imaging suites. We ascertained pregnancy or the peripartum period by event location – events occurring in delivery rooms, maternity wards and department of gynecology.

We excluded reports of RRT (rapid response team) calls for patients without cardiac arrest, for children under age 18 years, for patients with out-of-hospital cardiac arrest misclassified as IHCA, duplicate reports and empty reports. These were all identified during review of the written and electronic charts.

**Supplement 4.** List of study variables

This table lists the variables that were collected for this study. Variables that were computed using these variables are shown in the main paper.

| **Name** | **Type** | **Source** | **Values** |
| --- | --- | --- | --- |
| **Age** | continuous | medical chart | years |
| **Sex** | categorial | medical chart | male, female |
| **Last hemoglobin measured 24 hours prior to CPR (g/dl)** | continuous | medical chart | g/dL |
| **Lowest hemoglobin measured 48 hours prior to CPR (g/dl)** | continuous | medical chart | g/dL |
| **Charlson comorbidity index** | continuous | medical chart | number |
| **Illness category** | categorial | medical chart | cardiac, respiratory, metabolic, neurologic, infection, other |
| **Initial rhythm** | categorial | medical chart | VF/VT, PEA/Asystole |
| **Blood transfusion 72 hours prior to CPR** | categorial | medical chart | yes, no |
| **Date and Time of Event** | date | CPR report | date |
| **Event witnessed** | categorial | CPR report | yes, no |
| **Chest compressions** | categorial | CPR report | yes, no |
| **Shocked delivered** | categorial | CPR report | yes, no |
| **Adrenaline dose (mg)** | continuous | CPR report | mg |
| **Lidocaine given** | categorial | CPR report | yes, no |
| **Amiodarone given** | categorial | CPR report | yes, no |
| **Date and time CPR stopped** | date | CPR report | date |
| **ROSC** | categorial | CPR report | yes, no |
| **ECPR^1^** | categorial | medical chart | yes, no |
| **TTM^1^** | categorial | medical chart | yes, no |
| **Coronary angiography^1^** | categorial | medical chart | Yes, no |
| **Coronary reperfusion attempt^1^** | categorial | medical chart | PCI, none |
| **Survival to discharge** | categorial | medical chart | yes, no |
| **Discharge destination** | categorial | medical chart | home, rehabilitation, nursing home, ventilated ward, deceased |
| **Date and time of death** | date | medical chart | date |
| **Acute Myocardial Infraction** | categorial | medical chart | yes, no |
| **Old Myocardial Infarction** | categorial | medical chart | yes, no |
| **Congestive Heart Failure** | categorial | medical chart | yes, no |
| **Peripheral Vascular Disease** | categorial | medical chart | yes, no |
| **Acute Cerebrovascular Disease^2^** | categorial | medical chart | TIA or CVA with no or minor residua, major residua, none |
| **History of CVA^2^** | categorial | medical chart | minor CVA or TIA, hemiplegia, other residua, none |
| **Dementia** | categorial | medical chart | yes, no |
| **Pulmonary disease^2^** | categorial | medical chart | COPD, asthma, chronic pulmonary disease, none |
| **Connective Tissue Disease** | categorial | medical chart | yes, no |
| **Gastrointestinal Ulcer** | categorial | medical chart | yes, no |
| **Liver Disease^3^** | categorial | medical chart | Other, mild, moderate, severe, none |
| **Diabetes Mellitus^2^** | categorial | medical chart | uncomplicated, complicated, none |
| **Paraplegia** | categorial | medical chart | yes, no |
| **Renal Failure^2,4^** | categorial | medical chart | Mild, moderate, dialysis, none |
| **Solid Tumors^2^** | categorial | medical chart | primary, secondary, none |
| **Hematologic malignancy^2^** | categorial | medical chart | Lymphoma, Leukemia, none |
| **AIDS** | categorial | medical chart | yes, no |

^1^ Collected only for patients who achieved ROSC

^2^ for the analysis of individual disease states no division by severity or specific disease was used.

^3^ liver disease- Liver disease severity is as follows: other-liver abscess, liver metastases, elevated liver enzymes, fatty liver, shocky liver etc., mild- cirrhosis with no portal hypertension or chronic hepatitis, moderate- cirrhosis with portal hypertension but no variceal bleeding, severe- cirrhosis with portal hypertension and variceal bleeding. For the analysis of individual disease states the division was as follows: mild, moderate or severe- yes, other or none- no.

^4^ renal disease severity is as follows: mild- CR<3, moderate- CR>3, dialysis.

CPR- Cardio-pulmonary resuscitation. TTM- Targeted Temperature Management. ECMO- Extracorporeal Membrane Oxygenation. ROSC- Return of Spontaneous Circulation. CVA- cerebrovascular accident, TIA- Transient ischemic attack, COPD- chronic obstructive pulmonary disease, AIDS- acquired immunodeficiency syndrome. VF- ventricular fibrillation, VT- ventricular tachycardia, PEA- pulseless electrical activity. PCI- percutaneous coronary intervention. CR- creatinine.

**Supplement 5.** The ICD-9 codes used to identify comorbidities

| **Diagnosis** | **ICD-9 Code** | **Notes** |
| --- | --- | --- |
| **Acute Myocardial Infraction** | 410.00 - 410.92 |  |
| **Old Myocardial Infarction** | 412 |  |
| **Congestive Heart Failure** | 428.0 - 428.9 |  |
| **Peripheral Vascular Disease** | 443.9 |  |
| **Acute Cerebrovascular Disease** | 434.01 - 434.91, 435.9 | manual division by severity |
| **History Of Minor CVA or TIA** | V12.54 |  |
| **History Of Major CVA** | 430.0-438.9 | division for hemiplegia or other by hemiplegia column |
| **Dementia** | 290.0 - 290.9 |  |
| **COPD** | 496 |  |
| **Asthma** | 493.20 - 493.22, 493.9 |  |
| **Chronic Pulmonary Disease** | 516.0 - 516.32 |  |
| **Connective Tissue Disease** | 710.0-710.9 |  |
| **Ulcer Gastrointestinal** | 532.00 - 534.9 |  |
| **Liver Disease** | 570 - 573.9 | manual division by severity |
| **Diabetes Mellitus Without Complications** | 250.00- 250.03 | Division by severity using other columns of diabetes complications |
| **Diabetes Mellitus with Renal Manifestation** | 250.40 - 250.43 |  |
| **Diabetes Mellitus with Ophthalmic Manifestation** | 250.50 - 250.53 |  |
| **Diabetes Mellitus with Neurological Manifestation** | 250.60 - 250.63 |  |
| **Diabetes Mellitus with Peripheral Circulatory Disorder** | 250.70 - 250.73 |  |
| **Diabetic Foot** | 250.80 - 250.83 |  |
| **Late Effect of CVA - hemiplegia** | 438.2 |  |
| **Paraplegia** | 344.1 |  |
| **Renal Failure** | 585.9 | manual division by severity |
| **Solid Tumors** | 140 – 195 (primary)  196 - 198 (secondary)  V10.00-V10.99 (history of malignancy) | manual division by severity of those who were positive for history of malignancy |
| **Lymphoma** | 200.00 - 202.98 |  |
| **Leukemia** | 204.00 - 208.92 |  |
| **AIDS** | 42 |  |

ICD- International Classification of Diseases. CVA- cerebrovascular accident, TIA- Transient ischemic attack, COPD- chronic obstructive pulmonary disease, AIDS- acquired immunodeficiency syndrome.

**Supplement 6.** Data validation process

Shaare Zedek Medical Center maintains a database of ongoing prospectively collected CPR event data. For the purpose of this study, we first tested the database algorithm as follows. All the data extracted from the database regarding prior diseases and hemoglobin measurements were compared manually against patient files to check the robustness of the collecting algorithm. Incoherent and missing data were reported to the computer authority and the algorithm was edited to perfect the classification of each data column until the database presented correctly each comorbidity. Relevant data regarding the event was manually ascertained to check for coherence with medical charts and to complete missing data. Additional information regarding patients' characteristics was added and manually evaluated to check for robustness of the collecting algorithm and to fill-in missing data. Finally, all data were also tested for logic (e.g., age 999 or 150, illogical drug doses).

Examples of the cleaning process:

1. Hemoglobin measurements were initially extracted as text (instead of a number) causing the algorithm to identify measurements starting with 1 (e.g., 11, 10.2) as lower than result starting with a digit larger than 1 (e.g., 5.7, 8.6). The algorithm was adjusted to extract the correct measurement.
2. A mistake in the icd-9 codes for diabetes with renal complications caused a merge with the diagnosis of uncomplicated diabetes. This was revealed during the evaluation of the algorithm. The codes were correct, and the algorithm was examined for other technical mistakes in the icd-9 codes, that were not found.
3. We found that ICD-9 codes for present malignancy, old myocardial infarction and cerebrovascular disease initially did not cover all the cases who had these comorbidities based on their medical chart. Therefore, we added additional codes to fully cover these diagnoses.
4. Information on ROSC was lacking and had to be manually added.
5. Presence of an unmeasurable blood sample (due to insufficient sample volume or any other reason) in the 48 hours prior to the event were considered by the algorithm as the lower result. Thus, causing for several cases in which lowest hemoglobin measurement was absent but listed as measured. We examined those cases manually and added the measurable lowest result. In cases where the unmeasurable result was also the last, the proper measurable last result was also sought and reported.

**Supplement 7.** Patients missing data on hemoglobin

These patients were younger (mean age 72.5 ± 13.9 vs 76.6 ± 14.2, p=0.01), were more likely to have a cardiac cause for the arrest (52.8% vs 39.2%, P=0.017) and more commonly had a comorbidity of prior myocardial infarction (10.1% vs 4.9%, P=0.049). However, these patients comprised less than 10 % of eligible cases even before reduction of repeat resuscitations, therefore we proceeded with data analysis without these patients.

**Supplement 8.** Results based on the last hemoglobin measurement

- - 1. **Cohort characteristics**

Based on the last hemoglobin measurement, one third of the patients had anemia (39.2%, n=303), half of the patients did not have anemia (49.4%, n=382) and 11.4% (88) of the patients lacked a hemoglobin measurement from the 24 hours preceding the arrest (**supplement 16,17**). The mean last hemoglobin level was 10.63 ± 2.31 g/dL (median 10.3 g/dL, IQR 8.8-12.2 g/dL, range 4.6-19.4 g/dL). Last hemoglobin tertiles cut-offs were and 1^st^: ≤9.3 g/dL, 2^nd^: >9.4 g/dl and ≤11.5 g/dL, 3^rd^: >11.5 g/dL. When using this definition of anemia, anemic patients had higher prevalence of diabetes (41.6% vs 36.6%, P=0.036) and less acute CVA (1.7% vs 6.5%, P=0.002) (**supplement 17**). The cause of arrest differed between the groups. More anemic patients received a blood transfusion in the 72 hours preceding the arrest (23.8% vs 6.0%, P<0.001). Less anemic patients had an initial shockable rhythm (7.6% vs 13.4%, P=0.018) and anemic patients received less adrenaline (4.84 ± 3.32mg vs 5.78 ± 4.69mg, P=0.003). Less anemic patients underwent coronary angiography (0.7% vs 6.0% P<0.001) and a coronary reperfusion attempt after CPR (0.7% vs 4.5%, P=0.017). Finally, Anemia was not associated with ROSC (49.5% vs 51%, P=0.688) or survival to discharge (7.3% vs 10.7%, P=0.118) in unadjusted analyses (**supplement 16**).

- - 1. **Last hemoglobin and Charlson comorbidity index**

The CCI was correlated with the last hemoglobin (P=0.045, Pearson’s r -0.077) (**supplement 10b**). The mean CCI was 2.16 ± 1.91 in the anemic group vs 1.9 ± 1.85 in the non-anemic group (p=0.069). The mean CCIs in each of the three hemoglobin tertiles were: 1^st^: 2.14 ± 1.93, 2^nd^: 2.07 ± 1.83, 3^rd^: 1.82 ± 1.87 (P=0.172).

- - 1. **Survival to hospital discharge**
- **Univariable analysis**

We studied the association between last hemoglobin and survival to hospital discharge in three manners: First by comparing survival rates in anemic vs non anemic patients (primary outcome) (**supplement 16a**), then by comparing survival rates in the three hemoglobin tertiles (1^st^: 6.6%, 2^nd^: 9.5%, 3^rd^: 11.4%, P=0.209) and finally by comparing hemoglobin levels in survivors and non-survivors (**supplement 11**). We found no unadjusted association between last hemoglobin and survival to discharge when studied as survival rates between anemic and non-anemic patients and as survival rates between the three hemoglobin levels tertiles. We did find an unadjusted association between last hemoglobin and survival to hospital discharge when studied as hemoglobin levels between survivors and non-survivors.

- **Multivariable analysis**

Anemia based on the last hemoglobin, age, CCI, initial shockable rhythm, adrenaline dose and coronary angiography were entered into the multivariable analysis. The multivariable analysis included 313 patients and the Hosmer-Lemeshow test for goodness of fit had a p-value of 0.898. CCI, initial shockable rhythm, adrenaline dose and coronary angiography remained associated with survival to hospital discharge in the adjusted analysis. Anemia based on the last hemoglobin and age were not associated with survival to hospital discharge in the adjusted analysis (**supplement 18**).

**Supplement 9a.** Hemoglobin histogram - Last hemoglobin measured in the 24 hours preceding the arrest


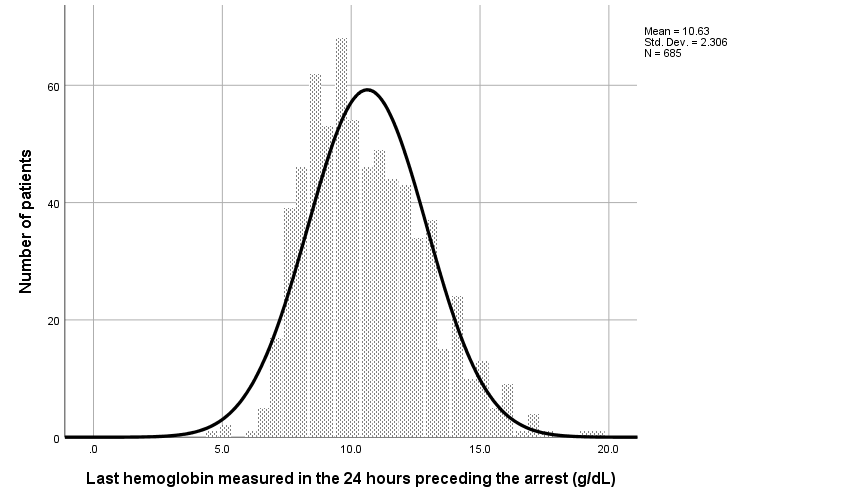


**Supplement 9b.** Hemoglobin histogram - Lowest hemoglobin measured in the 48 hours preceding the arrest


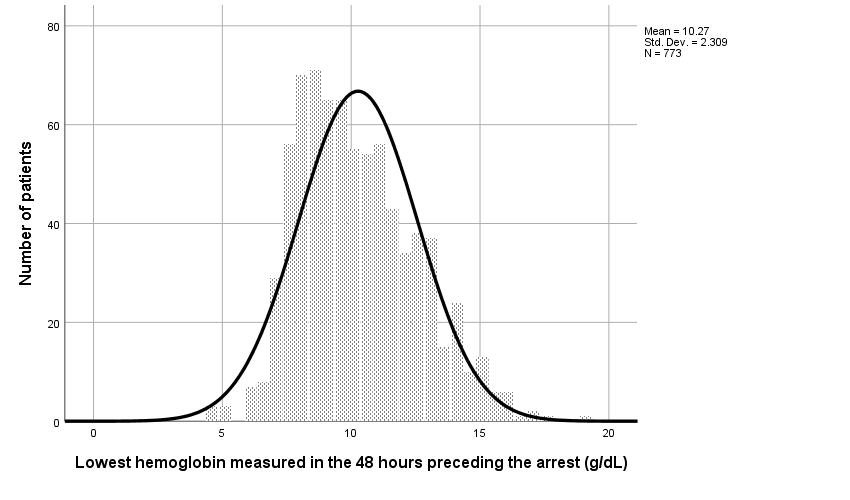


**Supplement 10a**. Plot of the association between CCI and lowest hemoglobin.


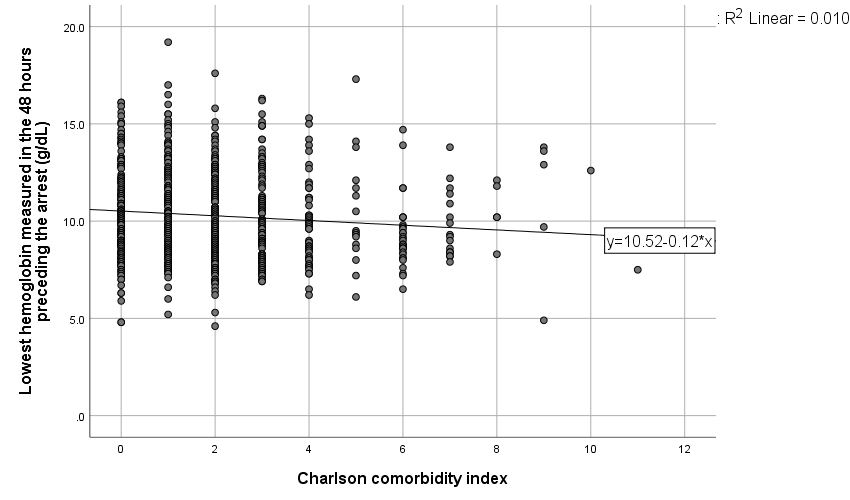


**Supplement 10b.** Plot of the association between CCI and last hemoglobin.


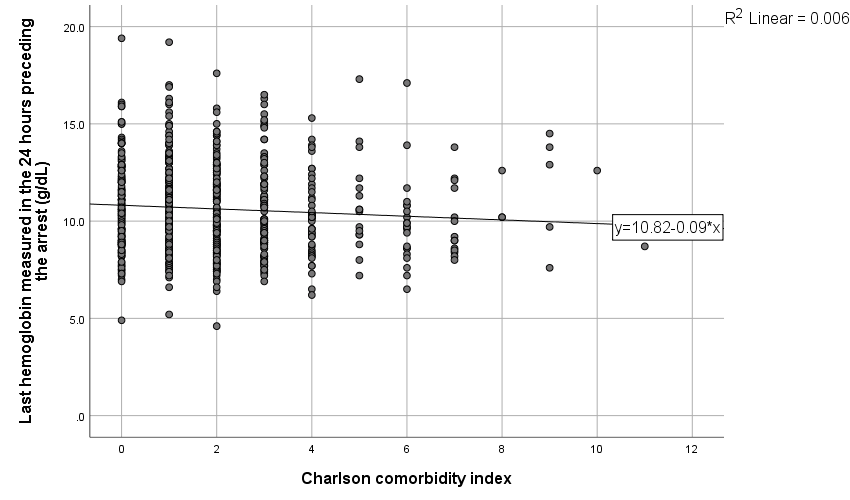


**Supplement 11a.** Patients’ characteristics divided by **survival to hospital discharge** – categorial variables. Values are presents as n (%).

| **Characteristics** | | | **Survivors (n=70)** | **Non-survivors (n=703)** | **P-Value** |
| --- | --- | --- | --- | --- | --- |
| **Male sex** | | | 42 (60) | 389 (55.3) | 0.454 |
| **Lowest hemoglobin anemia** | | | 30 (42.9) | 360 (51.2) | 0.183 |
| **Lowest hemoglobin tertiles** | | 1^st^ | 17 (24.3)  25 (35.7)  28 (40) | 246 (35)  232 (33)  225 (32) | 0.171 |
|  |  | 2^nd^ |  |  |  |
|  |  | 3^rd^ |  |  |  |
| **Last hemoglobin anemia** | | | 22 (31.4) | 281 (40) | 0.118 |
| **Last hemoglobin tertiles** | | 1^st^ | 15 (21.4)  22 (31.4)  26 (37.1) | 211 (30)  209 (29.7)  202 (28.7) | 0.209 |
|  |  | 2^nd^ |  |  |  |
|  |  | 3^rd^ |  |  |  |
| **Cause of arrest** | cardiac | | 27 (38.6) | 276 (39.3) | 0.135 |
|  | respiratory | | 33 (47.1) | 267 (38) |  |
|  | metabolic | | 2 (2.9) | 23 (3.3) |  |
|  | neurologic | | 0 (0) | 9 (1.3) |  |
|  | infection | | 2 (2.9) | 74 (10.5) |  |
|  | other | | 4 (5.7) | 19 (2.7) |  |
| **Initial shockable rhythm** | | | 20 (28.6) | 58 (8.3) | <0.001 |
| **Blood transfusion 72 hours prior to CPR** | | | 11 (15.7) | 88 (12.5) | 0.445 |
| **Event witnessed** | | | 42 (60) | 389 (55.3) | 0.345 |
| **Chest compressions** | | | 68 (97.1) | 695 (98.9) | 0.193 |
| **Shock delivered** | | | 25 (35.7) | 163 (23.2) | 0.021 |
| **Lidocaine given** | | | 3 (4.3) | 15 (2.1) | 0.182 |
| **Amiodarone given** | | | 9 (12.9) | 76 (10.8) | 0.417 |
| **ECPR** | | | 2 (2.9) | 8 (1.1) | 1.000 |
| **TTM** | | | 0 (0.0) | 2 (0.3) | 1.000 |
| **Coronary angiography** | | | 9 (12.9) | 17 (2.4) | 0.035 |
| **Coronary reperfusion attempt** | | | 7 (10.0) | 13 (1.8) | 0.068 |

CPR- cardio-pulmonary resuscitation. ECPR- extracorporeal cardiopulmonary resuscitation. TTM- targeted temperature management. Missing data- Last hemoglobin- 88 (11.4%), Cause of arrest – 37 (4.8%), Initial shockable rhythm – 32 (4.1%), Event witnessed – 20 (2.6%), Chest compressions – 1 (0.1%), Shock delivered – 2 (0.3%), Lidocaine and amiodarone – 26 (3.4%), ECPR, TTM, coronary angiography and coronary reperfusion attempt – 390 (50.5%).

**Supplement 11b.** Patients’ characteristics divided by **survival to hospital discharge** – Continuous variables.

| **Characteristics** | **Survivors (n=70)** | | | **Non-survivors (n=703)** | | | **P-Value** |
| --- | --- | --- | --- | --- | --- | --- | --- |
|  | Mean ± SD | Median (IQR) | Range | Mean ± SD | Median (IQR) | Range |  |
| **Age (years)** | 69.81 ± 15.42 | 71 (63-81) | 22-99 | 77.25 ± 139.92 | 81 (69-87) | 18-106 | <0.001 |
| **CCI** | 1.59 ± 1.29 | 1 (1-3) | 0-5 | 2.12 ± 1.96 | 2 (1-3) | 0-11 | 0.002 |
| **Lowest hemoglobin (g/dL)** | 10.8 ± 2.4 | 10.6 (9.0-12.1) | 6.6-17.6 | 10.2 ± 2.3 | 9.9  (8.4-11.7) | 4.6-19.2 | 0.055 |
| **Last hemoglobin (g/dL)** | 11.2 ± 2.5 | 11.2  (9.4-12.6) | 6.6-17.6 | 10.6 ± 2.3 | 10.2  (8.8-12.1) | 4.6-19.4 | 0.042 |
| **Adrenaline (mg)** | 2.89 ± 1.86 | 3 (1-4) | 0-8 | 5.61 ± 4.16 | 5 (3-7) | 0-36 | <0.001 |

CCI- Charlson comorbidity index. SD- standard deviation. IQR- interquartile range.

Missing data- Adrenaline – 43 (5.6%), Last hemoglobin – 88 (11.4%).

**Supplement 12.** Comorbidities of patients. Divided by **survival to hospital discharge**. Values are presented as n (%).

| **Comorbidities** | **Survivors (n=70)** | **Non-survivors (n=703)** | **P-Value** |
| --- | --- | --- | --- |
| **Acute MI** | 13 (18.6) | 85 (12.1) | 0.120 |
| **Old MI** | 2 (2.9) | 36 (5.1) | 0.567 |
| **CHF** | 26 (37.1) | 263 (37.4) | 0.965 |
| **PVD** | 1 (1.4) | 21 (3) | 0.712 |
| **Acute CVA** | 1 (1.4) | 34 (4.8) | 0.358 |
| **History of CVA** | 8 (11.4) | 99 (14.1) | 0.540 |
| **Dementia** | 3 (4.3) | 54 (7.7) | 0.300 |
| **Pulmonary disease** | 7 (10) | 66 (9.4) | 0.867 |
| **Connective Tissue Disease** | 0 (0) | 6 (0.9) | 1.000 |
| **Gastrointestinal ulcer** | 1 (1.4) | 7 (1) | 0.534 |
| **Liver disease** | 2 (2.9) | 14 (2) | 0.649 |
| **Diabetes mellitus** | 31 (44.3) | 278 (39.5) | 0.440 |
| **Paraplegia** | 0 (0) | 0 (0) |  |
| **Renal failure** | 10 (14.3) | 138 (19.6) | 0.278 |
| **Solid tumors** | 6 (8.6) | 113 (16.1) | 0.097 |
| **Hematologic malignancy** | 0 (0) | 26 (3.7) | 0.158 |
| **AIDS** | 0 (0) | 0 (0) |  |

MI – myocardial infarction. CHF- congestive heart failure. PVD- peripheral vascular disease. CVA- cerebrovascular accident. AIDS- acquired immune deficiency syndrome.

**Supplement 13.** Adjusted odds ratios of the independent variable for survival to hospital discharge (OR>1) in several sensitivity analyses. Each row represents a different model.

| **Variable** | | **univariable** | | | **multivariable** | | |
| --- | --- | --- | --- | --- | --- | --- | --- |
|  |  | OR | 95% CI | P-Value | OR | 95% CI | P-Value |
| **Controlled for:** Age, CCI, Initial shockable rhythm, Adrenaline dose and Coronary angiography | | | | | | | |
| **Lowest hemoglobin (mg/dL)** | | 1.105 | 0.998-1.224 | 0.056 | 1.119 | 0.980-1.278 | 0.097 |
| **Lowest hemoglobin tertiles** | 1^st^ | 0.555 | 0.296-1.042 | 0.067 | 0.482 | 0.210-1.106 | 0.085 |
|  | 2^nd^ | 0.866 | 0.490-1.531 | 0.620 | 1.004 | 0.465-2.168 | 0.993 |
|  | 3^rd^ | Ref | Ref | Ref | Ref | Ref | Ref |
| **Last hemoglobin (mg/dL)** | | 1.118 | 1.004-1.244 | 0.042 | 1.105 | 0.963-1.268 | 0.154 |
| **Last hemoglobin tertiles** | 1^st^ | 0.552 | 0.284-1.073 | 0.080 | 0.442 | 0.182-1.074 | 0.071 |
|  | 2^nd^ | 0.818 | 0.449-1.490 | 0.511 | 0.811 | 0.357-1.841 | 0.616 |
|  | 3^rd^ | Ref | Ref | Ref | Ref | Ref | Ref |
| **Controlled for:** Age, Solid tumors, Initial shockable rhythm, Adrenaline dose and Coronary angiography | | | | | | | |
| **Lowest hemoglobin anemia** | | 0.715 | 0.435-1.173 | 0.183 | 0.710 | 0.369-1.364 | 0.304 |
| **Last hemoglobin anemia** | | 0.651 | 0.379-1.119 | 0.118 | 0.633 | 0.310-1.294 | 0.210 |
| **Controlled for:** Age, Solid tumors, Initial shockable rhythm and Adrenaline dose | | | | | | | |
| **Lowest hemoglobin anemia** | | 0.715 | 0.435-1.173 | 0.183 | 0.712 | 0.390-1.301 | 0.269 |
| **Last hemoglobin anemia** | | 0.651 | 0.379-1.119 | 0.118 | 0.577 | 0.296-1.124 | 0.106 |

OR- odds ratio, CI- confidence interval, CCI- Charlson comorbidity index.

**Supplement 14.** Survival to hospital discharge and ROSC rates between anemic and non-anemic patients based on lowest and last hemoglobin measurements in subgroup analysis of males, females and patients that did not receive blood transfusions in the 72 hours preceding their arrets.

|  |  | **Lowest hemoglobin** | | | **Last hemoglobin** | | |
| --- | --- | --- | --- | --- | --- | --- | --- |
|  |  | **Anemia** | **No anemia** | **P-value** | **Anemia** | **No anemia** | **P-value** |
| **Survival to hospital discharge** | Males | 8.0% | 11.6% | 0.213 | 7.5% | 12.4% | 0.109 |
|  | Females | 7.2% | 9.1% | 0.530 | 7.08% | 8.7% | 0.590 |
|  | No blood transfusions | 7.0% | 10.2% | 0.136 | 6.5% | 10.6% | 0.090 |
| **ROSC** | Males | 51.3% | 49.3% | 0.669 | 50.0% | 53.6% | 0.484 |
|  | Females | 48.2% | 48.9% | 0.901 | 48.8% | 48.0% | 0.882 |
|  | No blood transfusions | 47.4% | 48.9% | 0.684 | 46.3 | 51.3% | 0.242 |

ROSC- return of spontaneous circulation. Missing data- Last hemoglobin – 88 (11.4%).

**Supplement 15**. Return Of Spontanous Circulation

- - 1. **Unadjusted association between lowest hemoglobin and ROSC**

No unadjusted association was found between lowest hemoglobin and ROSC. We studied this association in three manners: First by comparing ROSC rates in ROSC and non-ROSC patients (**table 1a**), then by comparing ROSC rates in the three hemoglobin levels tertiles (1^st^: 51.0%, 2^nd^: 48.6%, 3^rd^: 49.0%, p=0.852) and finally by comparing hemoglobin levels in ROSC and non- ROSC patients (**supplement 19b**).

- - 1. **Unadjusted association between lowest hemoglobin and ROSC**

No unadjusted association was found between last hemoglobin and ROSC. We studied this association in three manners: First by comparing ROSC rates in ROSC and non-ROSC patients (**supplement 16a**), then by comparing ROSC rates in the three hemoglobin levels tertiles (1^st^: 51.8%, 2^nd^: 50.6%, 3^rd^: 48.7%, P=0.801) and finally by comparing hemoglobin levels in ROSC and non-ROSC patients (**supplement 19a**)**.**

- - 1. **Other factors associated with ROSC – univariable analysis**

Patients who achieved ROSC were younger (**supplement 19b**) (74.05 ± 15.06 vs 79.06 ± 12.871, P<0.001), had more diabetes (45.2% vs 34.9%, P=0.003) and congestive heart failure (40.5% vs 34.4%, P=0.079) and had less renal failure (16.2% vs 22.1%, P=0.038), solid tumors (39.5% vs 51.4%, P=0.017), dementia (38.6% vs 61.4%, P=0.086) and connective tissue disease (**supplement 20**) (0% vs 1.5%, P=0.015). Patients who achieved ROSC also had more blood transfusion in the 72 hours prior to CPR (15.1% vs 10.5%, P=0.054), were more likely to be witnessed at the time of arrest (61.1% vs 50.5%, P=0.001), resultantly had more initial shockable rhythms (**supplement 19a**) (13.6% vs 6.7%, P=0.001), were given shocks more often (30.0% vs 18.7%, P<0.001) and received less adrenaline (4.97 ± 4.03 vs 5.78 ± 4.1, P=0.001).

- - 1. **Multivariable analysis**
       1. **Adjusted association between anemia based on the lowest hemoglobin and ROSC**

Anemia based on the lowest hemoglobin, age, CCI, blood transfusion, witnessed event, initial rhythm and adrenaline dose were entered into the multivariable analysis. The multivariable analysis included 685 patients and the Hosmer-Lemeshow test for goodness of fit had a p-value of 0.102. Age, blood transfusion, witnessed event, initial rhythm and adrenaline dose remained associated with ROSC in the adjusted analysis. Anemia based on the lowest hemoglobin and CCI were not associated with ROSC in the adjusted analysis (**supplement 21a**).

- - 1. **Adjusted association between anemia based on the last hemoglobin and ROSC**

Anemia based on the last hemoglobin, age, CCI, blood transfusion, witnessed event, initial rhythm and adrenaline dose were entered into the multivariable analysis. The multivariable analysis included 603 patients and the Hosmer-Lemeshow test for goodness of fit had a p-value of 0.149. Age, CCI, initial rhythm and adrenaline dose remained associated with ROSC in the adjusted analysis. Anemia based on the lowest hemoglobin, blood transfusion and witnessed event were not associated with ROSC in the adjusted analysis (**supplement 21b**).

- - 1. **Sensitivity analysis**

Sensitivity analysis of the independent variable (i.e., hemoglobin) by entering hemoglobin levels as those in ROSC and non-ROSC groups and by entering hemoglobin divided to tertiles, showed no adjusted association with ROSC.

Sensitivity analysis of the potential confounders (i.e., comorbidities) was done by entering individual comorbidities that showed an unadjusted association with ROSC into the multivariable analysis instead of CCI. Hence, congestive heart failure, dementia, diabetes, renal failure and solid tumors were entered. Connective tissue disease was not entered despite showing unadjusted association with ROSC since none of the six patients with this comorbidity achieved ROSC. There was no association between anemia and ROSC in this adjusted analysis.

Sensitivity analysis results were consistent for both lowest and last hemoglobin (**supplement 22**).

- - 1. **Subgroup analysis**

We found no unadjusted association between anemia and ROSC in males, females and patients that did not receive blood transfusion in the 72 hours preceding their arrest subgroups. These results were consistent based on both lowest and last hemoglobin measurements (**supplement 14**).

**Supplement 16a.** Patients’ characteristics. **Anemia was defined** **based on last hemoglobin in the 24 hours preceding the arrest** – categorial variables. Values are presents as n (%).

| **Characteristics** | | **no anemia (n=382)** | **anemia (n=303)** | **P-Value** |
| --- | --- | --- | --- | --- |
| **Male sex** | | 209 (54.7) | 174 (57.4) | 0.477 |
| **Cause of arrest** | cardiac | 163 (42.7) | 106 (35) | 0.029 |
|  | respiratory | 141 (36.9) | 117 (38.6) |  |
|  | metabolic | 6 (1.6) | 17 (5.6) |  |
|  | neurologic | 5 (1.3) | 4 (1.3) |  |
|  | infection | 42 (11) | 30 (9.9) |  |
|  | other | 9 (2.4) | 12 (4) |  |
| **Initial shockable rhythm** | | 51 (13.4) | 23 (7.6) | 0.018 |
| **Blood transfusion 72 hours prior to CPR** | | 23 (6) | 72 (23.8) | <0.001 |
| **Event witnessed** | | 226 (59.2) | 161 (53.1) | 0.075 |
| **Chest compressions** | | 379 (99.2) | 297 (98) | 0.311 |
| **Shock delivered** | | 104 (27.2) | 67 (22.1) | 0.115 |
| **Lidocaine given** | | 11 (2.9) | 7 (2.3) | 0.619 |
| **Amiodarone given** | | 49 (12.8) | 30 (9.9) | 0.205 |
| **ROSC** | | 195 (51) | 150 (49.5) | 0.688 |
| **ECPR** | | 7 (1.8) | 3 (1.0) | 0.523 |
| **TTM** | | 0 (0.0) | 1 (0.3) | 0.435 |
| **Coronary angiography** | | 23 (6.0) | 2 (0.7) | <0.001 |
| **Coronary reperfusion attempt** | | 17 (4.5) | 2 (0.7) | 0.003 |
| **Survival to discharge** | | 41 (10.7) | 22 (7.3) | 0.118 |
| **Discharge destination** | home | 19 (5.0) | 6 (2.0) | 0.090 |
|  | rehabilitation | 11 (2.9) | 4 (1.3) |  |
|  | nursing home | 3 (0.8) | 1 (0.3) |  |
|  | ventilated ward | 6 (1.6) | 8 (2.6) |  |
|  | Deceased | 341 (89.3) | 281 (92.7) |  |

CPR- cardio-pulmonary resuscitation. ROSC- return of spontaneous circulation. ECPR- extracorporeal cardiopulmonary resuscitation. TTM- targeted temperature management. Missing data (out of 685 patients with a measurement of last hemoglobin)- Cause of arrest – 33 (4.8)%, Initial shockable rhythm 29 (4.2%), Event witnessed – 20 (2.9%), Chest compressions – 1 (0.1%), Shock delivered – 2 (0.3%), Lidocaine and amiodarone – 24 (3.5%), ECPR, TTM, coronary angiography, coronary reperfusion attempt – 340 (49.6%), discharge destination – 5 (0.7%).

**Supplement 16b.** Patients’ characteristics. **Anemia was defined** **based on last hemoglobin in the 24 hours preceding the arrest** – Continuous variables.

| **Characteristics** | **no anemia (n=382)** | | | **anemia (n=303)** | | | **P-Value** |
| --- | --- | --- | --- | --- | --- | --- | --- |
|  | Mean ± SD | Median (IQR) | Range | Mean ± SD | Median (IQR) | Range |  |
| **Age (years)** | 77.22 ± 14.05 | 80 (69-87) | 22-106 | 75.3 ± 14.45 | 77 (68-85) | 18-101 | 0.079 |
| **CCI** | 1.9 ± 1.85 | 2 (1-3) | 0-10 | 2.16 ± 1.91 | 2 (1-3) | 0-11 | 0.069 |
| **Adrenaline (mg)** | 5.78 ± 4.69 | 5 (3-7) | 0-36 | 4.84 ± 3.32 | 4 (3-6) | 0-30 | 0.003 |

CCI- Charlson comorbidity index. SD- standard deviation. IQR- interquartile range.

Missing data (out of 685 patients with a measurement of last hemoglobin)- Adrenaline – 40 (5.8%).

**Supplement 17.** Comorbidities of patients. **Anemia was defined** **based on last hemoglobin in the 24 hours preceding the arrest**. Values are presented as n (%).

| **Comorbidities** | **no anemia (n=382)** | **anemia (n=303)** | **P-Value** |
| --- | --- | --- | --- |
| **Acute MI** | 90 (13.1) | 56 (14.7) | 0.186 |
| **Old MI** | 36 (5.3) | 24 (6.3) | 0.176 |
| **CHF** | 251 (36.6) | 149 (39) | 0.150 |
| **PVD** | 20 (2.9) | 12 (3.1) | 0.699 |
| **Acute CVA** | 30 (4.4) | 25 (6.5) | 0.002 |
| **History of CVA** | 97 (14.2) | 50 (13.1) | 0.366 |
| **Dementia** | 52 (7.6) | 33 (8.6) | 0.245 |
| **Pulmonary disease** | 66 (9.6) | 40 (10.5) | 0.405 |
| **Connective Tissue Disease** | 5 (0.7) | 3 (0.8) | 1.000 |
| **Gastrointestinal ulcer** | 7 (1) | 5 (1.3) | 0.472 |
| **Liver disease** | 13 (1.9) | 10 (2.6) | 0.121 |
| **Diabetes mellitus** | 275 (40.1) | 140 (36.6) | 0.036 |
| **Paraplegia** | 0 (0) | 0 (0) |  |
| **Renal failure** | 122 (17.8) | 60 (15.7) | 0.106 |
| **Solid tumors** | 96 (14) | 48 (12.6) | 0.220 |
| **Hematologic malignancy** | 23 (3.4) | 9 (2.4) | 0.102 |
| **AIDS** | 0 (0) | 0 (0) |  |

MI – myocardial infarction. CHF- congestive heart failure. PVD- peripheral vascular disease. CVA- cerebrovascular accident. AIDS- acquired immune deficiency syndrome.

**Supplement 18.** Multivariable analysis for survival to hospital discharge (OR>1) – **Anemia was defined** **based on last hemoglobin in the 24 hours preceding the arrest**.

N=313

Goodness of fit p-value = 0.898

| **Variable** | **univariable** | | | **multivariable** | | |
| --- | --- | --- | --- | --- | --- | --- |
|  | OR | 95% CI | P-Value | OR | 95% CI | P-Value |
| **Age** | 0.970 | 0.956-0.984 | <0.001 | 0.993 | 0.973-1.014 | 0.534 |
| **CCI** | 0.839 | 0.719-0.979 | 0.026 | 0.751 | 0.591-0.956 | 0.020 |
| **Initial shockable rhythm** | 4.581 | 2.681-8.779 | <0.001 | 3.664 | 1.558-8.618 | 0.003 |
| **Adrenaline (mg)** | 0.663 | 0.575-0.764 | <0.001 | 0.671 | 0.564-0.799 | <0.001 |
| **Coronary angiography** | 2.569 | 1.094-6.033 | 0.035 | 7.512 | 2.177-25.920 | 0.001 |
| **Last hemoglobin anemia** | 0.651 | 0.379-1.119 | 0.118 | 0.605 | 0.294-1.245 | 0.172 |

OR- odds ratio, CI- confidence interval, CCI- Charlson comorbidity index.

**Supplement 19a.** Patients’ characteristics divided by **ROSC** – categorial variables. Values are presents as n (%).

| **Characteristics** | | | **ROSC (n=383)** | **No ROSC (n=390)** | **P-Value** |  |
| --- | --- | --- | --- | --- | --- | --- |
| **Male sex** | | | 217 (56.7) | 214 (54.9) | 0.617 |  |
| **Lowest hemoglobin anemia** | | | 195 (50.9) | 195 (50) | 0.799 |  |
| **Lowest hemoglobin tertiles** | | 1^st^ | 134 (35)  125 (32.6)  124 (32.4) | 129 (33.1)  132 (33.8)  129 (33.1) | 0.852 |  |
|  |  | 2^nd^ |  |  |  |  |
|  |  | 3^rd^ |  |  |  |  |
| **Last hemoglobin anemia** | | | 150 (39.2) | 153 (39.2) | 0.688 |  |
| **Last hemoglobin tertiles** | | 1^st^ | 117 (30.5)  117 (30.5)  111 (29) | 109 (27.9)  114 (29.2)  117 (30) | 0.801 |  |
|  |  | 2^nd^ |  |  |  |  |
|  |  | 3^rd^ |  |  |  |  |
| **Cause of arrest** | cardiac | | 161 (42) | 142 (36.4) | 0.231 |  |
|  | respiratory | | 152 (39.7) | 148 (37.9) |  |  |
|  | metabolic | | 14 (3.7) | 11 (2.8) |  |  |
|  | neurologic | | 3 (0.8) | 6 (1.5) |  |  |
|  | infection | | 30 (7.8) | 46 (11.8) |  |  |
|  | other | | 14 (3.7) | 9 (2.3) |  |  |
| **Initial shockable rhythm** | | | 52 (13.6) | 26 (6.7) | 0.001 |  |
| **Blood transfusion 72 hours prior to CPR** | | | 58 (15.1) | 41 (10.5) | 0.054 |  |
| **Event witnessed** | | | 234 (61.1) | 197 (50.5) | 0.001 |  |
| **Chest compressions** | | | 378 (98.7) | 385 (98.7) | 0.751 |  |
| **Shock delivered** | | | 115 (30.0) | 73 (18.7) | <0.001 |  |
| **Lidocaine given** | | | 11 (2.9) | 7 (1.8) | 0.303 |  |
| **Amiodarone given** | | | 48 (12.5) | 37 (9.5) | 0.150 |  |

CPR- cardio-pulmonary resuscitation. ROSC- return of spontaneous circulation. Missing data- Last hemoglobin- 88 (11.4%), Cause of arrest – 37 (4.8%), Initial shockable rhythm – 32 (4.1%), Event witnessed – 20 (2.6%), Chest compressions – 1 (0.1%), Shock delivered – 2 (0.3%), Lidocaine and amiodarone – 26 (3.4%).

**Supplement 19b.** Patients’ characteristics divided by **ROSC** – Continuous variables.

| **Characteristics** | **ROSC (n=383)** | | | **No ROSC (n=390)** | | | **P-Value** |
| --- | --- | --- | --- | --- | --- | --- | --- |
|  | Mean ± SD | Median (IQR) | Range | Mean ± SD | Median (IQR) | Range |  |
| **Age (years)** | 74.05 ± 15.06 | 77 (65-85) | 18-101 | 79.06 ± 12.87 | 82 (72-88) | 21-106 | <0.001 |
| **CCI** | 2.01 ± 1.81 | 2 (1-3) | 0-11 | 2.14 ± 2.01 | 2 (1-3) | 0-9 | 0.137 |
| **Lowest hemoglobin (g/dL)** | 10.3 ± 2.4 | 9.9  (8.5-11.7) | 4.8-19.2 | 10.3 ± 2.3 | 10  (8.6-12.0) | 4.6-16.5 | 0.860 |
| **Last hemoglobin (g/dL)** | 10.7 ± 2.4 | 10.3  (8.8-12.1) | 6.2-19.4 | 10.6 ± 2.2 | 10.3  (8.8-12.2) | 4.6-17.1 | 0.605 |
| **Adrenaline (mg)** | 4.97 ± 4.03 | 4 (2-6) | 0-30 | 5.78 ± 4.10 | 5 (3-7) | 0-36 | 0.007 |

CCI- Charlson comorbidity index. SD- standard deviation. IQR- interquartile range.

Missing data- Adrenaline – 43 (5.6%), Last hemoglobin – 88 (11.4%).

**Supplement 20.** Comorbidities of patients. Divided by **ROSC**. Values are presented as n (%).

| **Comorbidities** | **ROSC (n=383)** | **No ROSC (n=390)** | **P-Value** |
| --- | --- | --- | --- |
| **Acute MI** | 55 (14.4) | 43 (11) | 0.164 |
| **Old MI** | 15 (3.9) | 23 (5.9) | 0.203 |
| **CHF** | 155 (40.5) | 134 (34.4) | 0.079 |
| **PVD** | 12 (3.1) | 10 (2.6) | 0.634 |
| **Acute CVA** | 18 (4.7) | 17 (4.4) | 0.820 |
| **History of CVA** | 46 (12) | 61 (15.6) | 0.144 |
| **Dementia** | 22 (5.7) | 35 (9) | 0.086 |
| **Pulmonary disease** | 39 (10.2) | 34 (8.7) | 0.486 |
| **Connective Tissue Disease** | 0 (0) | 6 (1.5) | 0.031 |
| **Gastrointestinal ulcer** | 5 (1.3) | 3 (0.8) | 0.502 |
| **Liver disease** | 10 (2.6) | 6 (1.5) | 0.295 |
| **Diabetes mellitus** | 173 (45.2) | 136 (34.9) | 0.003 |
| **Paraplegia** | 0 (0) | 0 (0) |  |
| **Renal failure** | 62 (16.2) | 86 (22.1) | 0.038 |
| **Solid tumors** | 47 (12.3) | 72 (18.5) | 0.017 |
| **Hematologic malignancy** | 9 (2.3) | 17 (4.4) | 0.121 |
| **AIDS** | 0 (0) | 0 (0) |  |

MI – myocardial infarction. CHF- congestive heart failure. PVD- peripheral vascular disease. CVA- cerebrovascular accident. AIDS- acquired immune deficiency syndrome.

**Supplement 21a.** multivariable analysis for ROSC (OR>1) – **Anemia was defined** **based on lowest hemoglobin in the 48 hours preceding the arrest**.

N=685

Goodness of fit p-value = 0.102

| **Variable** | **univariable** | | | **multivariable** | | |
| --- | --- | --- | --- | --- | --- | --- |
|  | OR | 95% CI | P-Value | OR | 95% CI | P-Value |
| **Age** | 0.974 | 0.964-0.985 | <0.001 | 0.973 | 0.961-0.9850 | <0.001 |
| **CCI** | 0.966 | 0.897-1.040 | 0.362 | 0.924 | 0.850-1.004 | 0.062 |
| **Blood transfusion 72 hours**  **prior to CPR** | 1.519 | 0.991-2.329 | 0.054 | 1.712 | 1.038-2.823 | 0.035 |
| **Witnessed event** | 1.625 | 1.214-2.174 | 0.001 | 1.469 | 1.066-2.023 | 0.019 |
| **Initial shockable rhythm** | 2.210 | 1.347-3.624 | 0.001 | 2.038 | 1.157-3.589 | 0.014 |
| **Adrenaline (mg)** | 0.950 | 0.914-0.987 | 0.009 | 0.923 | 0.886-0.963 | <0.001 |
| **Lowest hemoglobin anemia** | 1.037 | 0.782-1.375 | 0.799 | 1.040 | 0.746-1.450 | 0.816 |

OR- odds ratio, CI- confidence interval, CCI- Charlson comorbidity index. CPR- cardiopulmonary resuscitation.

**Supplement 21b.** multivariable analysis for ROSC (OR>1) – **Anemia was defined** **based on last hemoglobin in the 24 hours preceding the arrest**

N=603

Goodness of fit p-value = 0.149

| **Variable** | **univariable** | | | **multivariable** | | |
| --- | --- | --- | --- | --- | --- | --- |
|  | OR | 95% CI | P-Value | OR | 95% CI | P-Value |
| **Age** | 0.974 | 0.964-0.985 | <0.001 | 0.973 | 0.960-0.986 | <0.001 |
| **CCI** | 0.966 | 0.897-1.040 | 0.362 | 0.903 | 0.825-0.989 | 0.027 |
| **Blood transfusion 72 hours**  **prior to CPR** | 1.519 | 0.991-2.329 | 0.054 | 1.589 | 0.957-2.638 | 0.074 |
| **Witnessed event** | 1.625 | 1.214-2.174 | 0.001 | 1.398 | 0.993-1.968 | 0.055 |
| **Initial shockable rhythm** | 2.210 | 1.347-3.624 | 0.001 | 2.169 | 1.202-3.912 | 0.010 |
| **Adrenaline (mg)** | 0.950 | 0.914-0.987 | 0.009 | 0.924 | 0.884-0.965 | <0.001 |
| **Last hemoglobin anemia** | 0.940 | 0.695-1.271 | 0.688 | 0.914 | 0.643-1.298 | 0.614 |

OR- odds ratio, CI- confidence interval, CCI- Charlson comorbidity index. CPR- cardiopulmonary resuscitation.

**Supplement 22.** Adjusted odds ratios of the independent variable for ROSC (OR>1) in several sensitivity analyses. Each row represents a different model.

| **Variable** | | **univariable** | | | **multivariable** | | |
| --- | --- | --- | --- | --- | --- | --- | --- |
|  |  | OR | 95% CI | P-Value | OR | 95% CI | P-Value |
| **Controlled for:** Age, CCI, Blood transfusions 72 hours prior to CPR, Witnessed event,  Initial Shockable rhythm and Adrenaline dose | | | | | | | |
| **Lowest hemoglobin (mg/dL)** | | 0.995 | 0.936-1.057 | 0.860 | 1.004 | 0.932-1.082 | 0.920 |
| **Lowest hemoglobin tertiles** | 1^st^ | 1.081 | 0.765-1.526 | 0.660 | 1.031 | 0.685-1.554 | 0.883 |
|  | 2^nd^ | 0.985 | 0.696-1.394 | 0.933 | 1.018 | 0.690-1.502 | 0.929 |
|  | 3^rd^ | Ref | Ref | Ref | Ref | Ref | Ref |
| **Last hemoglobin (mg/dL)** | | 1.017 | 0.953-1.086 | 0.604 | 1.019 | 0.943-1.101 | 0.637 |
| **Last hemoglobin tertiles** | 1^st^ | 1.131 | 0.783-1.635 | 0.511 | 1.153 | 0.750-1.773 | 0.516 |
|  | 2^nd^ | 1.082 | 0.750-1.560 | 0.674 | 1.110 | 0.735-1.676 | 0.619 |
|  | 3^rd^ | Ref | Ref | Ref | Ref | Ref | Ref |
| **Controlled for:** Age, CHF, Dementia, Diabetes mellitus, Renal failure, solid tumors,  Blood transfusions 72 hours prior to CPR, Witnessed event, Initial Shockable rhythm and Adrenaline dose | | | | | | | |
| **Lowest hemoglobin anemia** | | 1.037 | 0.782-1.375 | 0.799 | 1.021 | 0.727-1.432 | 0.905 |
| **Last hemoglobin anemia** | | 0.940 | 0.695-1.271 | 0.688 | 0.891 | 0.623-1.274 | 0.527 |

OR- odds ratio, CI- confidence interval, CCI- Charlson comorbidity index. CHF- congestive heart failure. CPR- cardiopulmonary resuscitation.
